# Molecular features of the cytotoxicity of an NHE inhibitor: Evidence of mitochondrial alterations, ROS overproduction and DNA damage

**DOI:** 10.1186/s12885-016-2878-9

**Published:** 2016-11-05

**Authors:** Francesca Aredia, Sebastian Czaplinski, Simone Fulda, A. Ivana Scovassi

**Affiliations:** 1 Istituto di Genetica Molecolare CNR, Via Abbiategrasso 207, 27100 Pavia, Italy; 2 Dipartimento di Biologia e Biotecnologie “L. Spallanzani”, Università di Pavia, Via Ferrata 9, 27100 Pavia, Italy; 3 Institute for Experimental Cancer Research in Pediatrics, Goethe-University, Komturstrasse 3a, 60528 Frankfurt, Germany

**Keywords:** Apoptosis, Autophagy, HMA, Mitochondria, NHE, PAR, RIPK3, ROS

## Abstract

**Background:**

NH exchangers (NHEs) play a crucial role in regulating intra/extracellular pH, which is altered in cancer cells, and are therefore suitable targets to alter cancer cell metabolism in order to inhibit cell survival and proliferation. Among NHE inhibitors, amiloride family members are commonly used in clinical practice as diuretics; we focused on the amiloride HMA, reporting a net cytotoxic effect on a panel of human cancer cell lines; now we aim to provide new insights into the molecular events leading to cell death by HMA.

**Methods:**

Colon cancer cell lines were treated with HMA and analysed with: morphological and cellular assays for cell viability and death, and autophagy; biochemical approaches to evaluate mitochondrial function and ROS production; *in situ* detection of DNA damage; molecular tools to silence crucial autophagy/necroptosis factors.

**Results:**

HMA affects cellular morphology, alters mitochondrial structure and function, causes an increase in ROS, which is detrimental to DNA integrity, stimulates poly(ADP-ribose) synthesis, activates RIPK3-dependent death and triggers autophagy, which is unable to rescue cell survival. These features are hot points of an intricate network of processes, including necroptosis and autophagy, regulating the homeostasis between survival and death.

**Conclusion:**

Our results allow the identification of multiple events leading to cell death in cancer cells treated with HMA. The here-defined intricate network activated by HMA could be instrumental to selectively target the key players of each pathway in the attempt to improve the global response to HMA. Our data could be the starting point for developing a newly designed targeted therapy.

## Background

Cancer cells have to reassess their own metabolism in order to sustain high proliferation rate requiring a continuous need of ATP; also the tumour microenvironment, which is characterised by peculiar features including hypoxia and acidosis, favours cancer cell proliferation [1]. Malignant cells counteract the persistent oxygen demand by turning the high glycolysis rate into anaerobic lactic acid fermentation (“Warburg effect”, from Otto Heinrich Warburg who first hypothesised this phenomenon [2]). Acidosis is managed through the exchanges between the tumour cell and microenvironment [3, 4], governed by membrane bound proton pumps/transporters and Na^+^/H^+^ exchangers (NHEs) [5]. The best characterised NHEs regulate the intracellular pH through the exchange of intracellular H^+^ with extracellular Na^+^, and, in turn, cause the alkalinisation of the cytosol that sustains cancer growth; being hyperactivated in cancer cells, they became potent targets having an impact on tumour development, growth and spread [5, 6].

Several NHE inhibitors were developed (*e.g*. 2-aminophenoxazine-3-one, compound 9 T, cariporide and amiloride) [3, 4]. We focused on the amiloride derivative HMA (5-(N,N-hexamethylene) amiloride), showing a peculiar physical feature, which is to be fluorescent under conventional UV excitation in an acid milieu [7]. We detected an intense cytoplasmic blue fluorescent signal in human Adult Retinal Pigmented Epithelium 19 (ARPE19) cells incubated for short times with HMA, followed by impaired cell survival [7, 8].

The interest toward HMA is legitimated by the evidence that it is poorly toxic to non-transformed breast cells and mouse mammary cells compared to transformed breast cancer cell lines and mouse mammary cancer cells, suggesting that HMA targets selectively cancer cells [9]. As for identifying the death pathways responsible for HMA cytotoxic effect, we and others [9, 10] previously reported that HMA affects cell survival by triggering caspase-independent paradigms of cell death in human cancer cell lines [9, 10], as it occurred also for other amiloride derivatives [11, 12]. In the present study, we characterised the molecular events regulating HMA cytotoxicity in colon cancer cells, reporting alterations in mitochondrial structure and function triggering ROS (reactive oxygen species) production, DNA damage and poly(ADP-ribose) synthesis. Finally, we investigated the possible involvement of necroptosis and autophagy in cancer cell response to HMA.

## Methods

### Cell culture

Colon carcinoma HCT-116 cells (from Dr. C.R. Boland [13]) were cultured in D-MEM supplemented with 10 % foetal calf serum (FCS), 4 mM glutamine, 2 mM Na pyruvate, 100 U/ml penicillin and 0.1 mg/ml streptomycin. Colon adenocarcinoma HT-29 cells (obtained from Dr. R. Supino [14]) were grown in McCoy medium supplemented as for D-MEM. Reagents were purchased from Thermo Fisher (Waltham, MA, USA) and Euroclone (Milano, Italy). Cells were grown as monolayer at 37 °C in humidified atmosphere containing 5 % CO_2_ in 75 cm^2^ flasks (Corning, Amsterdam, The Netherlands). At confluence, cells were trypsinised with trypsin-EDTA (stock solution 10X, Sigma-Aldrich, Saint Louis, MO, USA) diluted in PBS (Thermo Fisher).

### Cell treatments

Cell lines were treated with HMA (Sigma-Aldrich, stock solution 80 mM in DMSO) at concentrations ranging from 5 μM to 40 μM (accordingly to [10]) for the time periods indicated in the text. In some experiments, cells were treated with 15 mM NAC (N-acetyl cysteine, Sigma-Aldrich, stock solution 100 mM); 20 μM zVAD.fmk (Bachem, Heidelberg, Germany, stock solution 20 mM); 50 μM Necrostatin-1 (Biomol, Hamburg, Germany, stock solution 38.56 mM); 10 μM Olaparib (SelleckChem, Houston, TX, USA, stock solution 100 mM); 400 μM α-Tocopherol (Sigma-Aldrich, stock solution 22.36 mM). Parallel samples were incubated with 0.1 % DMSO, *i.e*. the same final concentration used with the highest drug concentration.

### Morphological analysis

Cells (either unfixed or being fixed with 4 % paraformaldehyde (PFA) in PBS) were analysed in bright field microscopy.

### Transmission electron microscopy

For transmission electron microscopy (TEM), cells were processed as follows. Some cells were fixed with 2.5 % (v/v) glutaraldehyde and 2 % (v/v) PFA in 0.1 M phosphate buffer; pH 7.4, at 4 °C for 1 h, washed and incubated with 3,3’ diaminobenzidine (DAB, Sigma-Aldrich) (2 mg/ml in 50 mM Tris-HCl; pH 7.6) under simultaneous irradiation with two 8 W Osram Blacklite 350 lamps for 2 h at room temperature (spectral emission range between 430 and 470 nm, thus being suitable for FITC excitation). The cells were then post-fixed with 1 % osmium tetroxide (Electron Microscopy Sciences, Hatfield, PA, USA) and 1.5 % potassium ferrocyanide (Sigma-Aldrich) at room temperature for 1 h, dehydrated with acetone and embedded in Epon (Agar Scientific, Assing, Monterotondo, Italy). Parallel samples, after aldehyde fixation, were incubated with DAB under light irradiation, dehydrated with ethanol and embedded in LR White resin. As negative controls, some samples were processed as described above but omitting both DAB incubation and exposure to the excitation light. Ultrathin sections were weakly stained with uranyl acetate (Agar Scientific). The samples were finally examined and photographed with a Zeiss EM 900 electron microscope at 80 KV (Carl Zeiss, Jena, Germany). The micrographs were then developed and digitalised [15].

### Cell death analysis

Cells were seeded at the density of 2x10^4^/well in 24-well plates. After 24 h, cells were treated with the selected drug. At the end of the treatment, the cells were rinsed with PBS and trypsinised, washed in binding buffer (0.1 mM Hepes, 2.5 mM CaCl_2_, 140 mM NaCl; pH 7.4) and incubated for 15 min with Annexin V-FITC (Milteny Biotech, Bergisch Gladbach, Germany, 3 % in binding buffer). Samples were then washed again in binding buffer, stained with PI (propidium iodide, Sigma-Aldrich, diluted 1:1000 in binding buffer) and immediately analysed by flow cytometry (FACSCanto II, BD Biosciences, Heidelberg, Germany).

### Western blot

The expression level of a panel of proteins in total extracts was analysed by Western blot according to procedures routinely used in the laboratory [10, 16] using the primary antibodies listed in Table 1. HRP conjugated secondary antibodies were from Jackson Immuno-Research (Suffolk, UK). Three independent experiments were carried out. Band quantification was performed using ImageJ software (https://imagej.nih.gov/ij/).

**Table 1:** Primary antibodies for western blot (diluted 1:1000)

ANTIGEN	ANTIBODY	SOURCE	COMPANY
Atg 7	2631	Rabbit	Cell Signaling
Beclin-1	3738	Rabbit	Cell Signaling
MLKL	GTX107538	Rabbit	Acris Antibodies
RIPK1	H207	Rabbit	Santa Cruz
RIPK3	IMG-5846A	Rabbit	Imgenex
α-tubulin	DM1A	Mouse	Sigma-Aldrich
γ-tubulin	TU-30	Mouse	Santa Cruz

### Indirect immunofluorescence

Cells were seeded on coverslips (16-mm diameter) in 12-well plates, at the density of 5x10^4^ cells/ml. After 24 h, cells were treated with the appropriate drug concentration for increasing times (up to 24 h). The following antigens were probed:

- ***p62***: cells were fixed in PFA (2 % in PBS) for 15 min on ice, kept overnight in 70 % ethanol at -20 °C, washed three times in PBS, incubated for 30 min with 5 % skim milk in PBS, then incubated in a humidified chamber with polyclonal antibody against p62 (diluted 1:100 in PBS, Enzo Life Science, Farmingdale, NY, USA) for 1 h at 37 °C, washed three times with PBS and incubated with secondary antibody (111-225-003, Cy2-conjugated anti-rabbit, Jackson Immuno-Research) diluted 1:50 in PBS, for 1 h at 37 °C. Coverslips were finally washed three times with PBS in the dark, incubated for 10 min with 0.2 μg/μl DAPI (Sigma-Aldrich) and washed again for 30 min in PBS. Slides were finally mounted with 10 μl of Mowiol.
- ***8***-***oxoG***: cells were fixed overnight in methanol/acetone 1:1 at -20 °C, incubated for 45 min with 2 N HCl in order to allow access of the antibody to the chromatin, then neutralised for 25 min with 0.1 mM sodium tetraborate; pH 8.0. Subsequently, samples were incubated with 1 % BSA (in PBS containing 0.2 % Tween-20) for 15 min and stained for 1 h with the anti-8-oxoG antibody (clone N45.1, JaICA, Shizuoka, Japan) diluted 1:300 in PBS containing 0.2 % Tween. Coverslips were then washed and incubated with the goat anti-mouse secondary antibody (488-labelled Dylight, KPL, Gaithersburg, MD, USA, diluted 1:100), for 30 min and finally for 30 min with a donkey anti-goat tertiary antibody (Alexa Fluor 488, Invitrogen, Molecular Probes) diluted 1:200 in PBS containing 0.2 % Tween. Coverslips were then processed as described above.
- ***γ***-***H2AX***: cells were incubated with the monoclonal antibody against γ-H2AX (JBW301, Merck Millipore, Milano, Italy; diluted 1:5000 in PBS) according to a procedure routinely used in our laboratory [17].
- ***GRP78***: cells were initially processed as described above for p62, incubated for 1 h at 37 °C in a humidified chamber with polyclonal antibody against GRP78 (Thermo Fisher, diluted 1:50 in PBS), washed three times with PBS and incubated with TRITC-conjugated anti-mouse secondary antibody (115-025-146; Jackson Immuno-Research, diluted 1:50 in PBS) for 1 h at 37 °C. Coverslips were processed as described above.
- ***Ubiquitin***: cells were processed as for p62, incubated in a humidified chamber with the polyclonal antibody against ubiquitin (diluted 1:100 in PBS [18]) for 1 h at 37 °C, washed three times with PBS and incubated with the TRITC-conjugated anti-rabbit secondary antibody (111-025-003, Jackson Immuno-Research, diluted 1:50 in PBS) for 1 h at 37 °C. Coverslips were processed as above.
- ***LC3***, ***mtHSP70 and poly***(***ADP***-***ribose***) immunostainings were performed as previously described [10, 16]. For all the immunofluorescence experiments, cells were observed using a fluorescence microscope Olympus BX51, equipped with a 60X objective. The images were acquired with a digital camera Camedia C4040 (Olympus, Tokyo, Japan); Adobe Photoshop was used as elaborating software. At least 100 cells per sample were counted in three independent experiments.

### Quantification of ROS

Cells were seeded in 24-well plates at the density of 2x10^4^/well. After 24 h, cells were treated with the appropriate drug for the selected time; thereafter, the fluorogenic dye DCFDA (dichlorofluorescein diacetate, Invitrogen, Molecular Probes, 10 μg/ml) was added to the medium for 30 min. After diffusion into the cell, DCFDA is deacetylated by cellular esterases to a non-fluorescent compound, which is later oxidized by ROS into 2’,7’-dichlorofluorescein (DCF). DCF is a highly fluorescent compound, which can be detected by fluorescence spectroscopy with maximum excitation and emission spectra of 495 nm and 529 nm, respectively. Finally, cells were trypsinised, resuspended in PBS and analysed by flow cytometry (FACSCanto II, BD Biosciences). Each experimental point was conducted in triplicate.

### Evaluation of mitochondrial membrane potential

To determine the mitochondrial membrane potential, cells were seeded in 24-well plates at the density of 2x10^4^/well, treated with the proper drug and then incubated with 50 nM TMRM (tetramethylrodamine methylester) (Invitrogen, Molecular Probes) for 10 min at 37 °C. Thereafter, samples were trypsinised, resuspended in 100 μl of PBS and immediately analysed by flow cytometry (FACSCanto II, BD Biosciences).

### RNA interference

For transient knockdown, cells seeded in 24-well plates (for death analysis) or in 6-well plates (for western blot analysis) at the density of 7.5x10^5^/well cells were reversely transfected with 5 nM Silencer select control siRNA (4390844) or targeting siRNA (s21741 and s21741 for RIPK3; s20650, s20651 and s20652 for Atg7; s16537 and s16538 for Beclin-1), using Lipofectamine RNAiMAX reagent and Opti-MEM medium according to manufacturers’ protocols. All reagents were purchased from Thermo Fisher.

### Statistical analysis

The statistical analysis (*t*-test) was performed using Excel Office 2011. From the results of three independent experiments, the average value ± standard deviation (S.D.) was calculated.

## Results

### HMA affects cell morphology

**Fig. 1:**
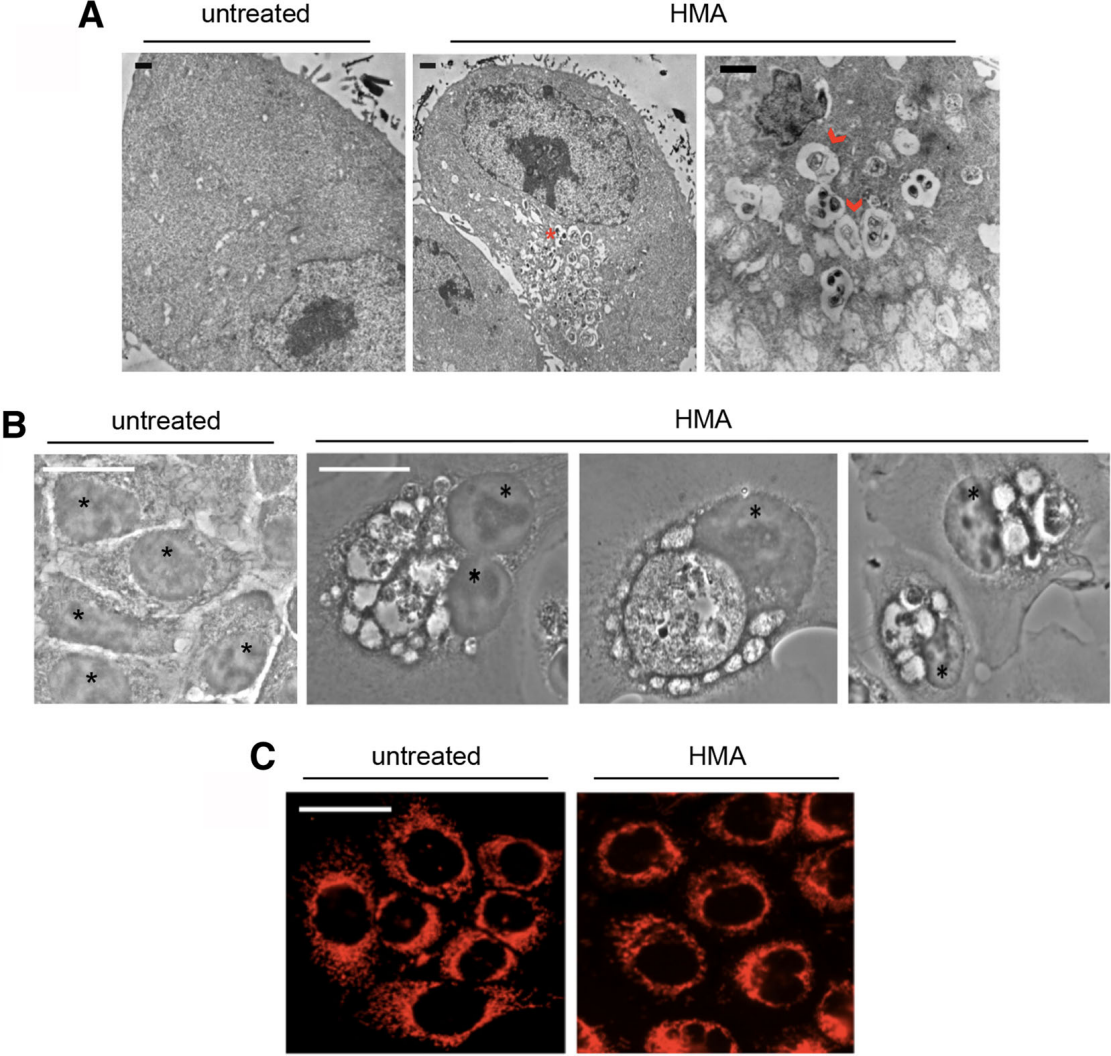
HMA affects cell morphology and mitochondria distribution. **a** TEM (transmission electron microscopy) analysis of diaminobenzidine (DAB) photo-oxidation in HCT-116 untreated cells and in cells treated with 20 μM HMA for 24 h. Red asterisk marks vesicles enclosing electron-dense regions within the cytoplasm; red arrowheads refer to vesicles containing intracellular debris. **b** Bright field microscopy images of HCT-116 cells untreated and treated with 20 μM HMA for 24 h; nuclei are marked with *. **c** Immunofluorescence analysis of mitochondrial HSP70 in untreated and HMA-treated (20 μM for 24 h) HCT-116 cells. Scale bar: 50 μm. A representative experiment out of three is shown

A preliminary survey of HMA effects on a panel of human cancer cell lines proved that HMA exerted a cytotoxic effect on three colon cancer cell lines, with an IC_50_ value of 22.30 μM for SW613-B3, 23.47 μM for HCT-116 and 32.19 μM for HT-29 cells [10]. Now, we aimed at depicting the molecular events leading to HMA cytotoxicity. First, we exploited the fluorescent properties of HMA [7] using the diaminobenzidine (DAB) photo-oxidation technique, which is based on the formation of an electron-dense osmiophilic product that precipitates in close proximity to the fluorophore, thereby allowing its ultrastructural detection [15, 19]. Electron microscopy analysis of HMA-treated HCT-116 colon carcinoma cells revealed electron-dense black spots within the cytoplasm, enclosed within vesicles (red*), which are present only as an effect of HMA, given that in untreated cells no evidence of roundish deposit of electron-dense products was found (Fig. 1a). In addition, in HMA-treated samples we detected some vesicles containing intracellular debris that can be likely associated to residual cisternae and resemble multilamellar bodies (red arrowhead) [20]. In fact, vacuole-like structures and swelling of intracellular structures were detected also by optical microscopy in the same conditions, exclusively in HMA-treated cells (Fig. 1b). On the whole, these observations suggest that HMA induces some mechanical stress due to the massive presence of vacuoles.

### Mitochondrial distribution and function are altered by HMA

After verifying that HMA had entered the cell and remained in the cytoplasmic compartment, where it promoted morphological alterations, we addressed its possible impact on the cytoplasmic organelles that play a master role in controlling cell metabolism, *i.e*. mitochondria. Their intracellular distribution was monitored by indirect immunofluorescence experiments using an antibody specific to the mitochondrial chaperone mtHSP70 (red fluorescence). We observed that mitochondria appeared to be rearranged in structure and localisation in HMA-treated HCT-116 cells: in untreated samples, mitochondria were distributed throughout the cytoplasm, while in HMA-treated cells they were detected as clustered outside the nuclear membrane (Fig. 1c).

### HMA stimulates ROS production

**Fig. 2:**
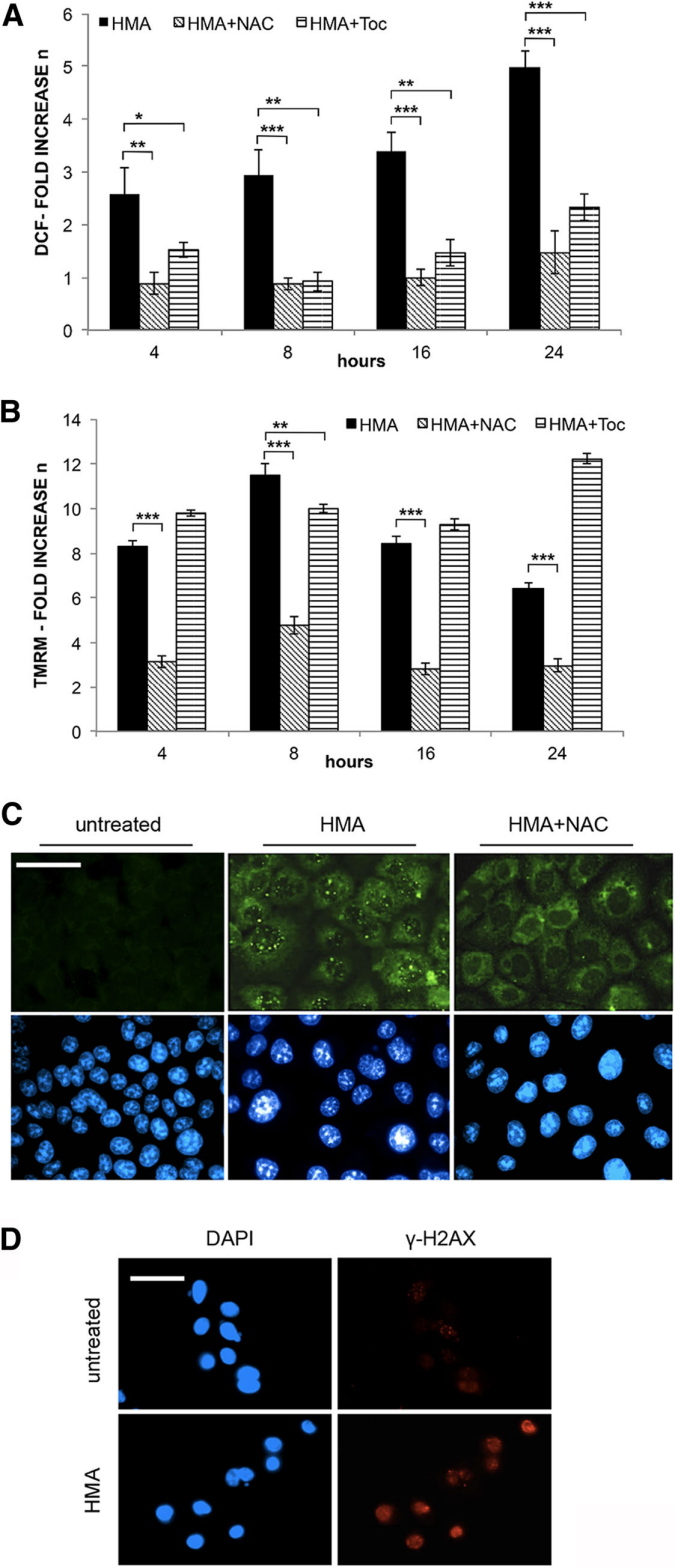
HMA stimulates ROS formation and mitochondrial membrane depolarization and affects DNA integrity. Effect of scavengers. **a** Quantification of ROS by cytofluorimetric analysis of DCF (dichlorofluorescein) fluorescence in HCT-116 cells treated for increasing times (4 h to 24 h) with HMA (30 μM) alone, or co-incubated with HMA and antioxidants (15 mM NAC or 400 μM α-Tocopherol, Toc) for the same time periods. **b** Measurement of mitochondrial membrane potential by cytofluorimetric analysis of TMRM (tetramethylrodamine methylester) fluorescence in HCT-116 cells treated as in (**a**). In **a** and **b**, data are expressed as fold increase with respect to untreated cells (mean ± s.d. calculated on three independent experiments). *P < 0.05, **P < 0.01, ***P < 0.001. **c** Detection of DNA damage by 8-oxoguanine immunostaining of untreated and HMA (20 μM, 24 h)-treated HCT-116 cells; parallel samples were incubated simultaneously with HMA and with 15 mM NAC. **d** Detection of γ-H2AX by immunostaining in untreated and HMA treated (20 μM, 24 h) HCT-116 cells. Scale bar: 50 μm. A representative experiment out of three is shown in panels (**c**) and (**d**), where nuclei were counterstained with DAPI (blue fluorescence)

As mitochondrial functions are strictly correlated with ROS production, we have measured the ROS level through a cytofluorimetric assay based on the use of the fluorogenic dye DCFDA that measures hydroxyl, peroxyl and other ROS within the cell; once diffused into the cell, it is deacetylated to a non-fluorescent compound, which is later oxidized by ROS into the highly fluorescent compound DCF [21]. In fact, as illustrated in Fig. 2a, in HMA-treated HCT-116 cells the quantification of DCF fluorescence intensity, which is proportional to ROS amount, showed a net time-dependent increase. When cells were cotreated for increasing times (up to 24 h) with well-known antioxidants, *i.e*. NAC (N-acetyl-L-cysteine) and α-Tocopherol (Toc), ROS levels were drastically reduced (Fig. 2a), indicating that both scavengers were effective in counteracting ROS production triggered by HMA.

### Mitochondrial membrane depolarization

To monitor mitochondrial transmembrane potential, we used TMRM, a lipophilic cation that normally penetrates the mitochondrial lipid bilayer and accumulates in the mitochondrial membrane emitting a fluorescence peak at 574 nm measurable by flow cytometry while it does not enter altered mitochondria, thus lowering the detectable intramitochondrial fluorescence. A sharp peak of fluorescence corresponds to cells with healthy mitochondria incorporating and accumulating the dye, whereas cells presenting depolarized (low fluorescence emission) or hyperpolarized (high fluorescence emission) mitochondria can be identified at the left and right side of the fluorescence peak of untreated cells, respectively. For each sample, the number of HCT-116 cells included in each class was recorded. No high fluorescence emission/hyperpolarization was observed; the ratio between cells with low (HMA-treated samples) and normal (untreated samples) fluorescence emission was calculated and reported as fold increase. As shown in Fig. 2b, HMA induced a net depolarization of the mitochondrial membrane yet after 4 h of treatment (8.33 folds), which increased even more at 8 h (11.5 folds) while a prolonged incubation allowed the cells to partially buffer mitochondrial membrane depolarization (8.44 and 6.44 fold at 16 h and 24 h, respectively). Similar results were obtained with the HT-29 colon cancer cell line (data not shown). NAC protected the cells from the HMA-induced depolarization, while no effect was observed with α-Tocopherol (Fig. 2b). This discrepancy could be due to the fact that the α-Tocopherol is a lipophilic cation that enters mitochondrial membranes only if mitochondria have an intact membrane potential, while the mechanism of action of the water-soluble NAC implies a rapid reaction with highly oxidising radicals.

### HMA affects DNA integrity

A high level of ROS is very dangerous for the cell, being a source of oxidation of DNA and organelles. We monitored the production of 7,8-dihydro-8-oxoguanine (8-oxoG), which is the most frequent oxidation product in both DNA and RNA and possibly contributes to various inflammatory processes and aging-related diseases [22]. HCT-116 cells were analysed by conventional *in situ* immunolabeling with a monoclonal antibody against 8-oxoG [23]. As shown in Fig. 2c, untreated cells were negative for the presence of 8-oxoG, while in all the cells treated for 24 h with 20 μM HMA, brilliant green fluorescent foci corresponding to the formation of 8-oxoG were clearly visible, confirming the presence of oxidised bases previously observed by the comet assay in HMA-treated cancer cells, thus supporting the postulated correlation between ROS production and base oxidation [10]. In parallel samples treated with NAC in combination with HMA, few foci were still detectable, possibly due to a low residual ROS amount (Fig. 2c).

The comet assay previously applied to HMA-treated cells showed a net increase of single- and double-strand breaks (SSBs and DSBs) [10]; here, we monitored the γ-H2AX form of the H2AX histone that is phosphorylated when DSBs are present in DNA [24]. In fact, as shown in Fig. 2d, a high fraction of HMA-treated cells (57.96 % ± 3.62), showed many red fluorescent nuclei (not visible in untreated cells), as expected in γ-H2AX positive cells. Together, these data support the notion that HMA was able to affect DNA integrity, possibly via ROS production.

### RIPK3 contributes to HMA-induced cell death

**Fig. 3:**
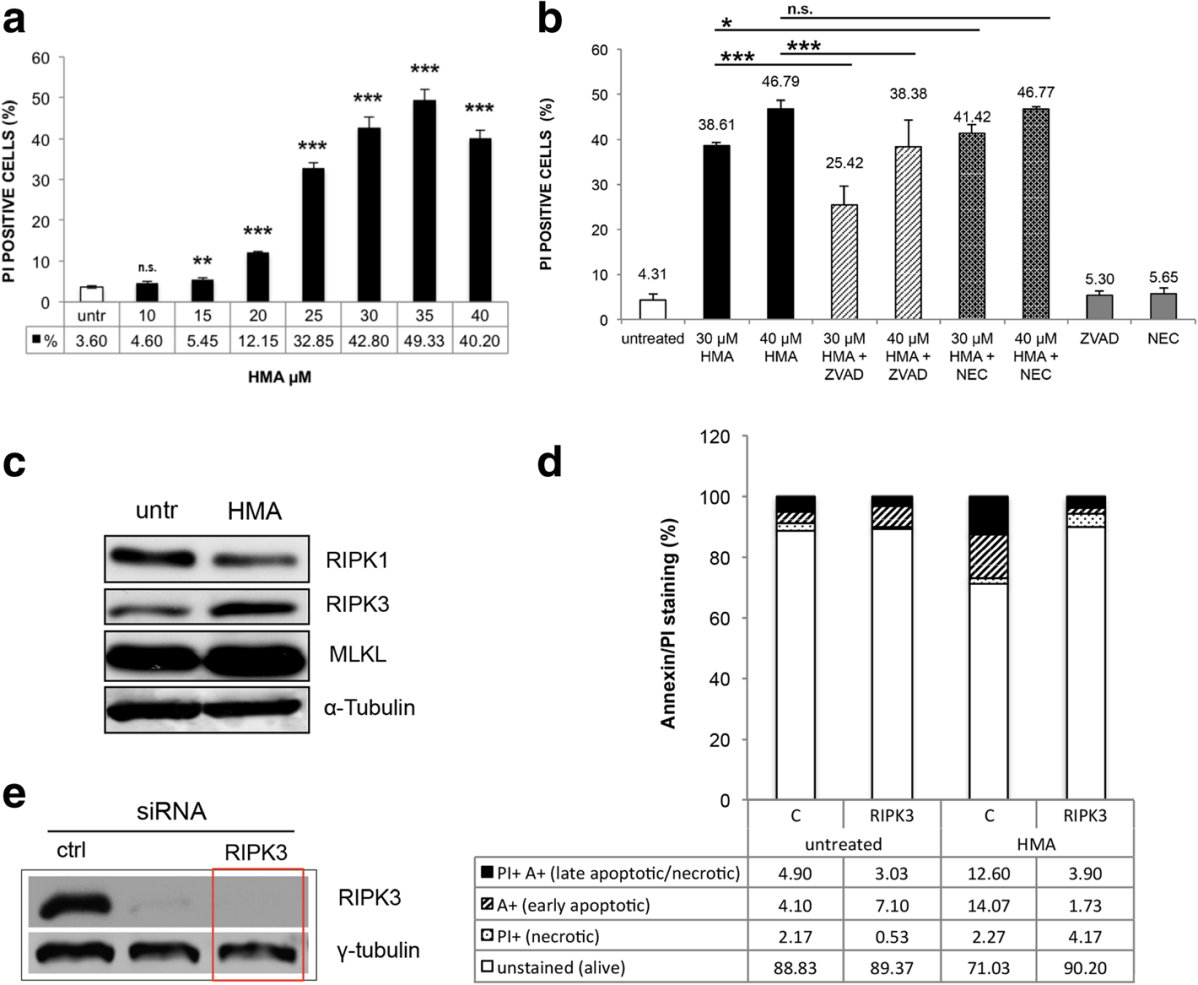
Cell death induced by HMA. Involvement of RIPK3. **a** PI (Propidium Iodide) permeability of untreated and HMA-treated (10-40 μM, 24 h) HCT-116 cells. Cytofluorimetric analysis allowed the quantification of PI^+^/dead cells detected on 10,000 events (mean ± s.d. calculated on three independent experiments). **P < 0.01, ***P < 0.001. **b** Cytofluorimetric analysis of untreated and HCT-116 cells treated with HMA alone (30 μM and 40 μM) or together with 20 μM pancaspase inhibitor zVAD.fmk or necroptosis inhibitor Necrostatin-1 (NEC, 50 μM); parallel samples were incubated with zVAD.fmk or NEC alone. Cells were stained with PI; values are expressed as percentage of PI positive events on 10,000 events recorded calculated on three independent experiments. *P < 0.05, ***P < 0.001, *n.s*. not significant. **c** Western blot analysis of necroptosis proteins RIPK1, RIPK3 and MLKL in HT-29 cells untreated and treated with 30 μM HMA for 24 h. α-Tubulin: loading control. **d** Effect of silencing of RIPK3 by siRNA on HT-29 cell survival after a 30-μM HMA treatment (for 24 h). Cell death was monitored by flow cytometry after Annexin V/PI staining; four fractions can be distinguished: PI^+^/A^+^: late apoptotic/necrotic cells; A^+^: early apoptotic cells; PI^+^: necrotic cells; unstained: alive cells. **e** Western blot analysis of RIPK3 in HT-29 cells after transfection with siRNAs; γ-tubulin was used as loading control; red rectangle refers to the siRNA used in **d**). A representative experiment out of three is shown in panels (**c**) and (**e**)

The presence of DNA damage, a high amount of ROS together with compromised mitochondria, as well as alterations in cell morphology after HMA treatment, could have an impact on cell viability. We stained cells with PI, which does not enter living cells, while it penetrates dying/dead cells, and analysed them by flow cytometry. HCT-116 cells treated with increasing concentrations of HMA (10-40 μM) for 24 h revealed a highly significant (*P* < 0.001) dose-dependent increase in the amount of PI^+^/dead cells, reaching about 50 % at 35 μM and remaining significant at 40 μM HMA (*P* < 0.001), even if the net number of PI^+^ cells declined to about 40 % (Fig. 3a). No effect on cell viability was recorded in samples incubated with the drug solvent DMSO (not shown).

How do HMA-treated cells die? We previously reported that HMA was unable to trigger the final steps of canonical apoptosis even if it promotes the activation of the initiator caspase 8 and 9 [10]. However, given that caspase 8 could be involved in a cross talk between apoptosis and other forms of death [25], here we used the pan-caspase inhibitor zVAD.fmk in combination with HMA. Cells treated with 20 μM zVAD.fmk alone showed a low amount of PI-permeable cells, similar to untreated samples. PI-stained cells after a 24-h treatment with 30 μM and 40 μM of HMA in the presence of zVAD.fmk (20 μM) revealed a significant (*P* < 0.001) decrease in the number of PI positive/dead cells compared to HMA treatment alone (Fig. 3b), accounting for 38.61 % ± 0.75 (30 μM HMA) and 25.42 % ± 4.16 after HMA/zVAD.fmk treatment. Analogously, for the 40 μM HMA concentration, the fraction of PI positive cells decreased from 46.79 % ± 1.76 to 38.38 % ± 5.92, highlighting a positive effect of the caspase inhibitor on cell viability and suggesting that initiator caspase 8 and 9 could be involved in other subroutes of death driven by HMA.

With this in mind, we investigated necroptosis, a death process where a role for caspase 8 has been described [26]. The possible involvement of necroptosis in the response to HMA was first investigated in HCT-116 cells by using the RIPK1 (receptor-interacting serine/threonine-protein kinase1) inhibitor Necrostatin-1 (NEC, 50 μM), which did not affect cell viability *per se* (Fig. 3b). When administered together with HMA (30 μM and 40 μM) for 24 h, NEC did not rescue HMA-induced cell death (Fig. 3b), thus suggesting that in HCT-116 cells RIPK1 is not involved in the cellular response to HMA, as already shown in breast cancer cells [9]. To go deeper into the necroptosis issue by addressing the impact of the other key regulator RIPK3, we used the HT-29 cell line, being HCT-116 cells characterised by a low expression of RIPK3 [27].

Western blot analysis of the expression of necroptosis effectors RIPK1 and 3 and MLKL (mixed lineage kinase domain-like) in untreated and HMA-treated HT-29 samples. We observed a modulation in response to the drug treatment, with an increase in RIPK3 and MLKL proteins in HMA-treated samples with respect to controls (1.60 and 1.97 fold, respectively; *P* < 0.01) (Fig. 3c); however, in this cell line an opposite trend was recorded for RIPK1 (0.60 fold decrease; *P* < 0.01). As reviewed by Lalaoui et al. [28], the requirement of RIPK1 in necroptosis is not absolute and cells lacking or expressing low levels of RIPK1 (as it is the case of HT-29 cells) undergo necroptosis by spontaneously increase the expression levels of RIPK3 and MLKL, as here observed.

Focusing on RIPK3 in HT-29 cells, we then used a different experimental approach, based on silencing of RIPK3. In HT-29 cells silenced for RIPK3 expression (Fig. 3e), the cell population characterised not only by PI permeability but also by late phosphatidylserine expression detected with Annexin V was decreased upon treatment with HMA, leading to an increase of the number of living cells from about 70 % to about 90 % (Fig. 3d). This suggests that the kinase RIPK3 is required, at least in part, for cell death induction by HMA.

### Autophagy modulates cell response to HMA

**Fig. 4:**
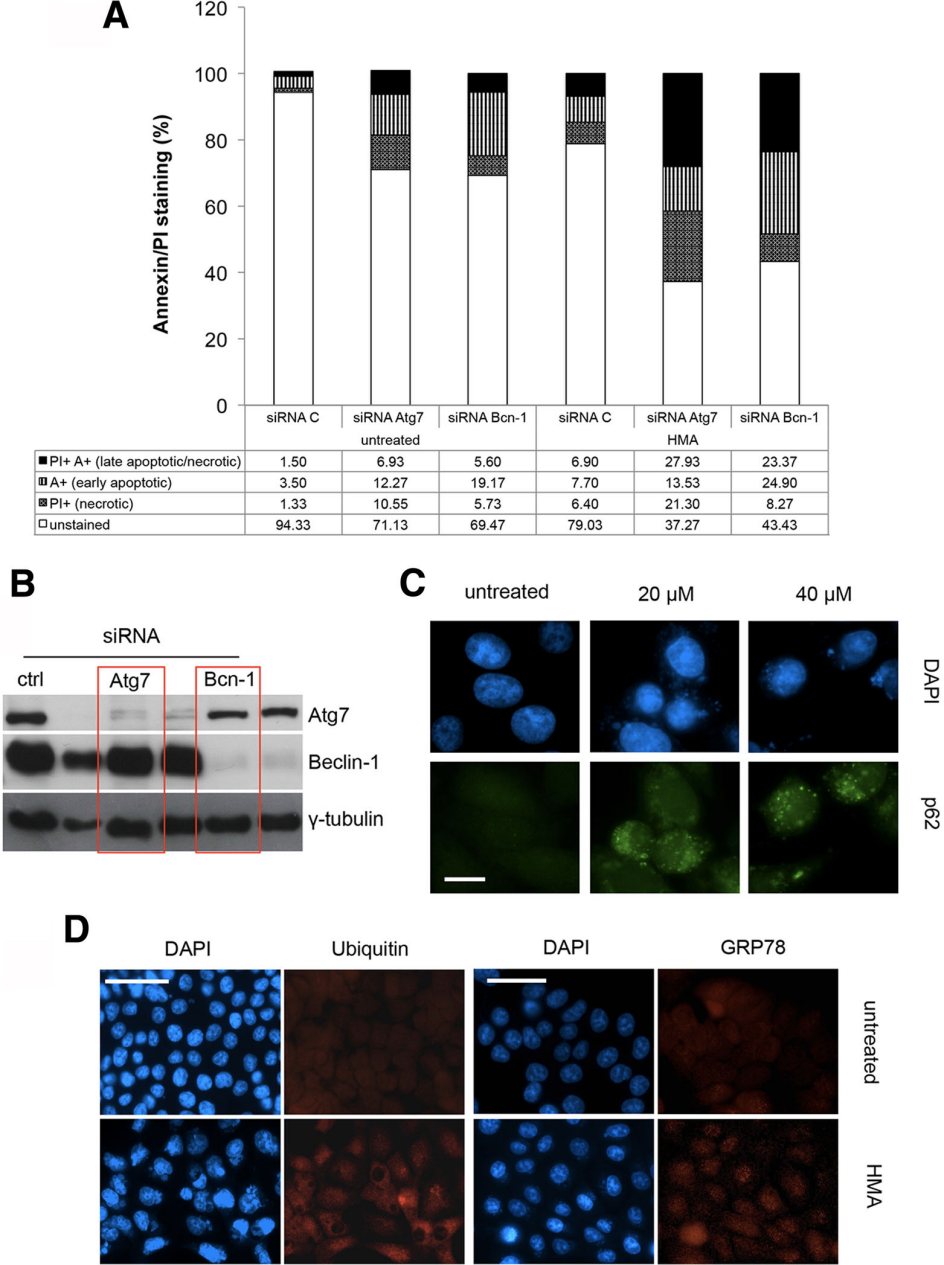
Activation of autophagy by HMA. Silencing of ATG7 and Beclin-1 by siRNA in HCT-116 cells. Impact of HMA on Ubiquitin and GRP78. **a** Effect of silencing of ATG7 and Beclin-1 on HCT-116 cell survival after a 30-μM HMA treatment (for 24 h). Cell death was monitored by flow cytometry after Annexin V/PI staining; four fractions can be distinguished: PI^+^/A^+^: late apoptotic/necrotic cells; A^+^: early apoptotic cells; PI^+^: necrotic cells; unstained: alive cells. **b** Western blot analysis of Atg7 and Beclin-1 in HCT-116 cells after transfection with siRNAs; γ-tubulin: loading control. Red rectangles refer to the siRNA used in **a**). **c** Immunofluorescence analysis of p62 (green fluorescence) in HCT-116 cells untreated and treated with 20 μM and 40 μM HMA for 24 h. Nuclei were counterstained with DAPI (blue fluorescence). Scale bar: 50 μm. A representative experiment out of three is shown. **d** Immunofluorescence analysis of Ubiquitin and GRP78 (red fluorescence) in HCT-116 cells untreated and treated with 20 μM HMA for 24 h; nuclei were counterstained with DAPI (blue fluorescence)

We previously detected some autophagy markers in HMA-treated colon carcinoma SW613-B3 cells [10], as also confirmed in breast cancer cells [9]; now, we addressed whether HMA drives a pro-survival or pro-death role of autophagy. To do this, we silenced Atg7 and Beclin 1 autophagy effectors (Fig. 4b) and then we analysed HCT-116 cell viability by Annexin V/PI staining. Both Atg7 and Beclin-1 silencing caused a decrease in unstained/alive cells of about 40 % upon HMA-treatment (from 79.03 % of control siRNA to 37.27 % for Atg7 and 43.43 % for Beclin-1), as well as enhanced cell death (Fig. 4a) supporting a protective role of autophagy in the presence of HMA-induced stress. However, autophagy was not completely efficient in rescuing cell viability after HMA treatment.

To address this intriguing point, we investigated the last events in the autophagy pathway. The final steps of autophagy are characterised by the sequestration of factors to be discarded through the action of the protein p62, also called sequestosome, which drives the cargo within autophagosomes, and is further degraded. In HMA-treated cells, p62 was detected as several spots (green fluorescence) and clustered both at nuclear and cytoplasmic level independently of the drug concentration (20 μM or 40 μM) (Fig. 4c). In fact, the analysis of the global ubiquitination level using an antibody against ubiquitin, revealed a low red fluorescent signal in untreated samples, while in HMA-treated cells several brilliant regions were visible (both in nuclear and cytoplasmic regions), possibly coincident with p62 distribution (Fig. 4d). Moreover, we analysed the UPR (unfolded protein response) marker GRP78 [29]: in control cells, GRP78 staining appeared as a low diffuse fluorescence, while in HMA-treated cells (20 μM) several specific dots were visible, indicating that GRP78 protein remained accumulated after the drug insult (Fig. 4d). Globally, our results point to a defect in the last phase of autophagy, leading to an impaired clearance of stress-damaged proteins and to an incomplete protection against HMA-induced insults.

### Poly(ADP-ribose) modulates HMA response

**Fig. 5:**
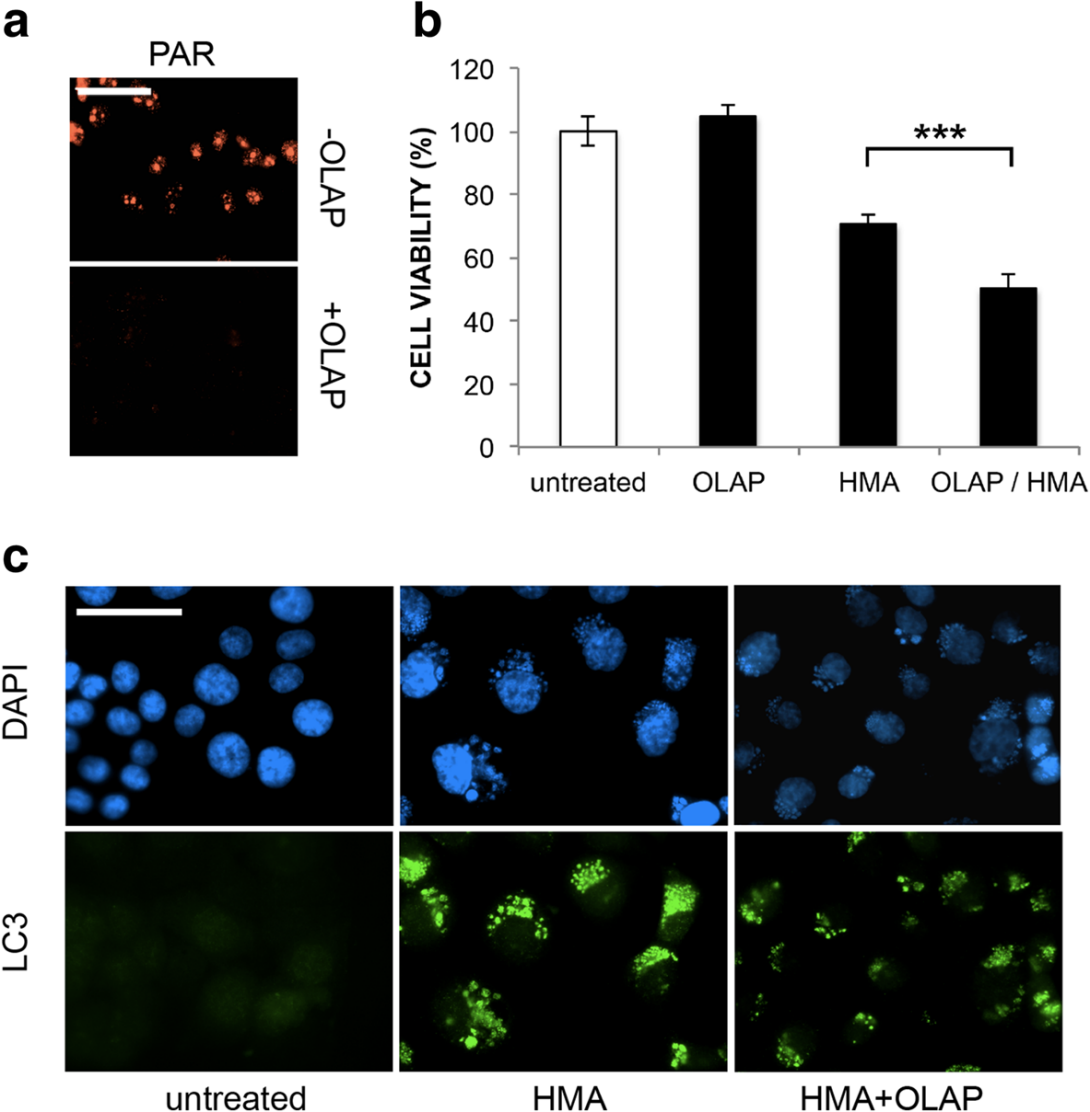
Effect of Olaparib on cell viability and autophagy. **a** Immunofluorescence detection of PAR (*red*) in HCT-116 cells treated for 24 h with 20 μM HMA without or with 10 μM Olaparib (OLAP). **b** MTT assay on samples as in (**a**) and in cells treated with OLAP only (10 μM for 24 h) or untreated; ***P < 0.001. **c** Immunofluorescence analysis of autophagy marker LC3 expression (green fluorescence) in HCT-116 cells under the above conditions; nuclei were counterstained with DAPI (blue fluorescence). Scale bar: 50 μm. A representative experiment out of three is shown

The evidence that HMA triggers DNA damage and oxidative stress conditions prompted us to investigate this effect on a cellular emergency reaction, *i.e*. poly(ADP-ribosylation), which we have already demonstrated to be activated by HMA [8, 10]. We addressed this issue in our experimental conditions by using the PARP inhibitor Olaparib (OLAP) under conditions abolishing PAR synthesis triggered by 20-μM HMA exposure, that are 10 μM for 24 h (Fig. 5a). First, we evaluated cell viability by the metabolic MTT assay, observing once more the inhibitory effect of HMA alone, causing a reduction of cell viability to 70.48 % ± 2.96 and, when combined to OLAP, decreasing to 50.24 % ± 4.74 (Fig. 5b), suggesting a net effect of the PARP inhibitor, so a role of PAR in modulating cell responses to HMA-treatment. Is PAR pro-survival function connected to autophagy? To answer this question, we monitored the autophagy marker LC3, generally overexpressed in HMA-treated cells [9, 10]; in samples co-treated with OLAP, a significant reduction of the LC3 signal with respect to the HMA treatment alone was observed (Fig. 5c), suggesting a possible requirement of PAR in the autophagy machinery [25, 30]. However, given the pleiotropic effects of PAR in cell metabolism, further experiments are required to better understand this issue.

## Discussion

Among the approaches used to manipulate the regulators of cancer cell homeostasis that favour proliferation and spread, often correlated with the so called Warburg effect [31], pH modulation through the targeting of the plasma membrane effectors has been exploited. In this context, amiloride, a clinically used potassium sparing diuretic, and derivatives have been used to promote intracytosolic acidification in cancer cells and ultimately counteract their survival and proliferation [32]; however, the mechanism of action leading to cancer cell death by these compounds is far from being elucidated.

We investigated the properties of the amiloride derivative HMA (5-(N,N- hexamethylene) amiloride), which was found to lower intracellular pH in rodent cells [33] with the consequent activation of acid DNases causing DNA degradation typical of caspase-independent paradigms of cell death [33]. In line with this pivotal paper, we previously described a cytotoxic effect of HMA on human Adult Retinal Pigmented Epithelium 19 (ARPE19) cells [8] and on a panel of human cancer cell lines [10], where HMA did not trigger canonical apoptosis but activated autophagy. In this respect, the recent paper by Rowson-Hodel et al. [9] confirmed that the cytotoxic effect of HMA on breast cancer cells was not due to classical caspase-dependent apoptosis but was triggered by a novel form of programmed necrosis.

In this study, we aimed to determine the molecular events leading to colon cancer cell death, which may happen through the destabilisation of organelles due to the intracytosolic localisation of HMA once entered the cell, as we have shown for the first time exploiting the fluorescent properties of HMA [7] and confirmed here by EM analysis (Fig. 1a). We focused on mitochondria, which act as the control unit of the cell being involved in the cell response to a wide range of insults [34], showing that in HMA-treated colon cancer cells they appeared to be relocalised to the perinuclear region (as recently reported in breast cancer cells [9]) and functionally impaired due to mitochondrial membrane depolarization. This is in agreement with the observations obtained in erythroleukemia cells treated with 5-(N-ethyl-N-isopropyl) amiloride [35] and in glioma cells with 5-benzylglycinyl-amiloride [36] and with the well-known inhibition of mitochondrial complex I activity by amilorides [37].

Based on the connection between dynamic variations in mitochondria, altered mitochondrial functions and increased generation of mitochondrial ROS [38], we observed a significant time-dependent increase in ROS, partially rescued by NAC, which counteracted also mitochondrial membrane depolarization. Our data, in line with the observation on breast cancer cells [9], raise an unresolved debate: are mitochondria the primary/exclusive source of ROS [39], or could ROS be originated from other organelles in the cell (*e.g*. ER or peroxisomes) and impact on mitochondria status/function? [40]. Remarkably, in agreement with the notion that a high ROS level can ultimately lead to DNA lesions [41], we found that HMA induces an expected increase in both guanine oxidation and histone H2AX phosphorylation. This first set of data allowed the identification of the early molecular events triggered by HMA in colon cancer cells.

Which pathway(s) leading to cell death can be triggered by mitochondrial structural/functional alterations, DNA damage and ROS accumulation? Despite the activation of initiator caspase 8 and 9, in our experimental conditions the canonical apoptosis pathway was not reaching the end, as both DNA ladder and PARP-1 proteolysis were absent [10]; the absence of PARP-1 proteolysis was recently described also in HMA-treated breast cancer cells [9]. The inability to end the apoptotic cascade is not intrinsic in colon cancer cells, having been reported that HCT-116 cells undergo canonical apoptosis after various drug treatments triggering ROS formation [42, 43]. However, the impact of autophagy in driving cell response could account for this feature, as it occurs in our context and in similar experimental conditions [44].

However, given that the pancaspase inhibitor zVAD.fmk failed to provide complete protection against HMA, as also reported in breast cancer cells [9], we considered necroptosis, another subroutine of programmed cell death [45, 46] that was recently shown to be triggered by amiloride derivatives in breast cancer [11] and glioma [12] cells. Although the inhibitor of RIPK1 Necrostatin-1 did not affect HMA cancer cell viability in our experimental conditions and also in a similar context [9], we found that the silencing of RIPK3 allowed a rescue in cell viability, indicating that this necroptosis factor (and not RIPK1) could be involved in cell death activation after HMA administration. The impact of a necrosis-like death pathway has been described in breast cancer cells [9], thus adding a new entry in the complex scenario of HMA response by cancer cells; this pathway was found to be independent of RIPK3 and MLKL expression level [9], while our data support their active role; these discrepant results can be explained by the different biological material used. Further investigations will clarify this issue.

To explore additional pathways leading to cell death through DNA damage and oxidative stress conditions, we focused on the accumulation of PAR (poly(ADP-ribose), a signaling molecule for AIF (apoptosis inducing factor) translocation from mitochondria that triggers nuclear DNA degradation during PAR/AIF-dependent cell death named parthanatos [47]. In fact, we previously demonstrated the activation of this pathway in colon cancer cells treated with HMA [10], and other groups confirmed our findings in breast [11] and glioma [12] cancer cells treated with 5-benzylglycinyl-amiloride and 5-glycinyl-amiloride. The real impact of PAR-dependent processes on the survival of amiloride-treated cancer cells has been addressed previously using the 1^st^-generation PAR inhibitor 3-aminobenzamide, showing that the reduction in PAR synthesis was ineffective in restoring cell viability [12]. However, here we used the last-generation compound Olaparib under conditions leading to the complete inhibition of PAR accumulation and we provide evidence that it was able to enhance the loss of cell viability after the combined administration with HMA.

Intriguingly, in HMA-treated cells we observed here an unexpected effect of Olaparib on the expression of the protein LC3, a marker of autophagy. The activation of autophagy (often mediated by mitochondrial ROS [48]) is necessary to degrade misfolded or damaged cell components, including mitochondria, thus promoting cancer cell survival. However, under extreme conditions, it can act as programmed cell death mechanism type II [49]. In our experimental conditions, autophagy played a pro-survival role in cancer cells treated with HMA, given that cell viability decreased, although only partially, when the crucial autophagy regulators Beclin-1 or ATG7 were silenced. Our data are in line with our previous evidence of a non-canonical form of HMA-driven autophagy [10] and with the results provided by Leon et al. [11] and Rowson-Hodel et al. [9] indicating that the induction of autophagy cannot account for the majority of HMA’s cytotoxic effects.

Why did autophagy fail in completely rescuing cancer cells from the early effects of HMA? We hypothesize that autophagosomes are unable to completely digest their content, resulting in a deadlocking of the autophagy machinery; in fact, electron microscopy images revealed that multilamellar membranes, residual of damaged organelles, are engulfed in membranes but not completely degraded. Our hypothesis was confirmed by the persistence of p62, ubiquitinated proteins and the ER stress marker GRP78 in HMA-treated cells, which are unable to efficiently discard the above proteins. Of note, multilamellar bodies could originate from lysosomal organelles that are possibly involved in the lysosome-dependent form of cell death recently identified in breast cancer cells treated with HMA [9].

## Conclusions

**Fig. 6:**
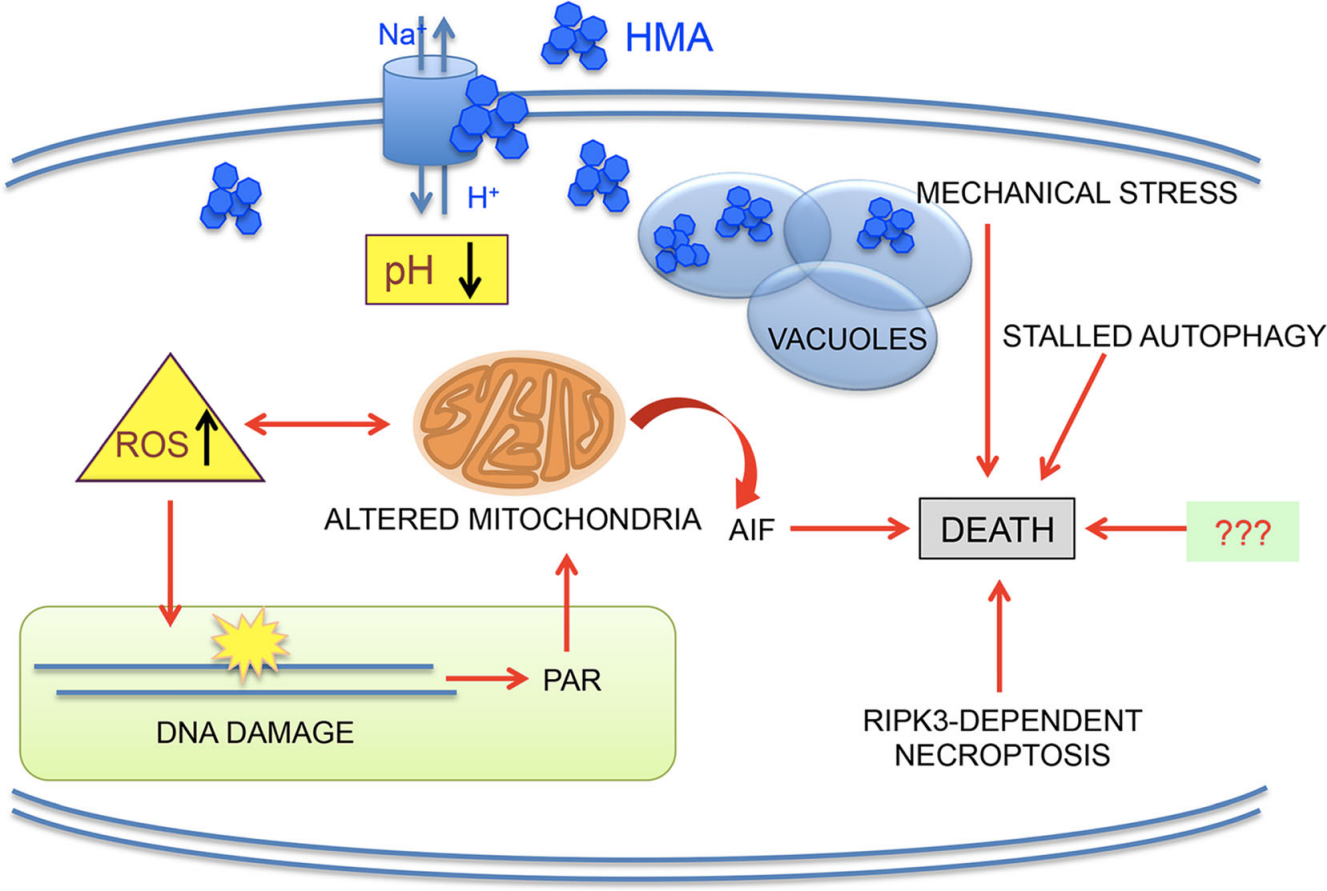
Multiple effects of HMA on colon cancer cell metabolism. HMA inhibits NHEs, thus lowering intracellular pH, enters the cell and induces mechanical stress and metabolic perturbations, which, in turn, stimulate ROS production, causing damage to mitochondria and DNA. DNA damage is then recognized by PARP-1 (poly(ADP-ribose) polymerase-1) that reacts to the dangerous situation by synthesizing high amounts of PAR (poly(ADP-ribose). PAR can act here as a signalling death molecule for mitochondrial AIF (apoptosis inducing factor) , which is then released from damaged mitochondria and, promotes cell death through the parthanatos process. After this cascade of events, autophagy is futile and its stall contributes to cell death; moreover, a pathway of death dependent on the necroptosis factor RIPK3 becomes activated. In addition to these factors/processes, other still unknown mechanisms (denoted as “???”) could be involved in HMA toxicity

In conclusion, as summarised in Fig. 6, we hypothesize that HMA enters the cell, lowers intracytosolic pH, causes cytosol vacuolization and organelle destabilization, so determining a mechanical stress, promotes ROS production affecting mitochondrial functions and DNA integrity and stimulates PAR synthesis, which triggers AIF-dependent parthanatos death.

After this cascade of events, autophagy is futile and its stall contributes to cell death; moreover, a pathway of death dependent on the necroptosis factor RIPK3 becomes activated. Each factor/process here considered could account only in part for HMA toxicity, thus other mechanisms (denoted as “???”) must be involved in the complex impact of HMA on cell survival, including lysosome and Ca^2+^-mediated death processes and impaired cell survival axes [9]. The knowledge of the molecular networks activated by HMA could be instrumental in identifying key players in the attempt to improve the global cancer cell response to HMA, possibly developing a newly designed targeted therapy.

## Abbreviations

AIF: Apoptosis inducing factor
DAB: 3,3’ diaminobenzidine
DAPI: 4’,6-diamidino-2-phenylindole
DCF: Dichlorofluorescein
DCFDA: Dichlorofluorescein diacetate
HMA: 5-(N,N-hexamethylene)amiloride
MLKL: Mixed lineage kinase domain-like
NAC: N-acetyl-L-cysteine
NHE: NH exchanger
PAR: Poly(ADP-ribose)
PFA: Paraformaldehyde
PI: Propidium iodide
RIPK: Receptor-interacting serine/threonine-protein kinase
TEM: Transmission electron microscopy
TMRM: Tetramethylrodamine methylester
UPR: Unfolded protein response
